# A 5‐Year Retrospective Morbidity Profile of School‐Aged Children at Ho Polyclinic, Volta Region, Ghana: Skin Conditions, Malaria, and Respiratory Illnesses as Leading Causes (2018–2022)

**DOI:** 10.1002/puh2.70347

**Published:** 2026-08-14

**Authors:** Anthoinette Adesope Ekuban, Frank Baiden

**Affiliations:** ^1^ Fred Newton Binka School of Public Health University of Health and Allied Sciences, Ho Volta Region Ghana

**Keywords:** Ghana, malaria, morbidity, respiratory conditions, school‐aged children, skin diseases

## Abstract

**Background:**

The disease burden among school‐aged children (5–17 years) in low‐ and middle‐income countries remains poorly characterized, with health policies predominantly focused on children under five. This study described the morbidity profile and examined associated demographic factors among school‐aged children attending a primary care facility in Ghana.

**Methods:**

A retrospective review of outpatient records was conducted at Ho Polyclinic in the Volta Region. Data from all consultations for children aged 5–17 years between January 2018 and December 2022 were extracted. The facility has a historical specialization in dermatological care because it was formally a leprosarium. Descriptive statistics summarized demographics and morbidity frequencies. Chi‐square tests assessed associations between age group, sex, and the three leading diagnostic categories.

**Results:**

Among 10,428 consultations (mean age 10.3 ± 4.0 years; 54.4% female), skin conditions (38.3%), malaria (23.0%), and respiratory conditions (16.1%) constituted 77.4% of all diagnoses. This pattern remained consistent across the 5‐year study period. No statistically significant associations were found between age group or sex and diagnosis of skin conditions (age *p* = 0.598; sex *p* = 0.611), malaria (age *p* = 0.176; sex *p* = 0.452), or respiratory conditions (age *p* = 0.839; sex *p* = 0.555).

**Conclusion:**

A consistent triad of infectious and environmental conditions dominates the morbidity profile of school‐aged children in this setting. The uniformity of this burden across demographic subgroups underscores the need for integrated, school‐based health interventions targeting skin health, malaria prevention, and respiratory hygiene to address this neglected demographic.

## Graphical abstract

Analysis of 10,428 outpatient consultations among school‐aged children attending Ho Polyclinic, Ghana (2018–2022), showed that three conditions accounted for 77.4% of all morbidity: skin conditions (38.3%), malaria (23.0%), and respiratory conditions (16.1%). The concentration of disease burden within these preventable and treatable conditions underscores the need for integrated school‐based health programs that combine health promotion, early case detection, preventive interventions, and timely treatment. Targeting these priority morbidities through coordinated school health services could substantially reduce the burden of disease and improve the health and educational outcomes of school‐aged children in Ghana.

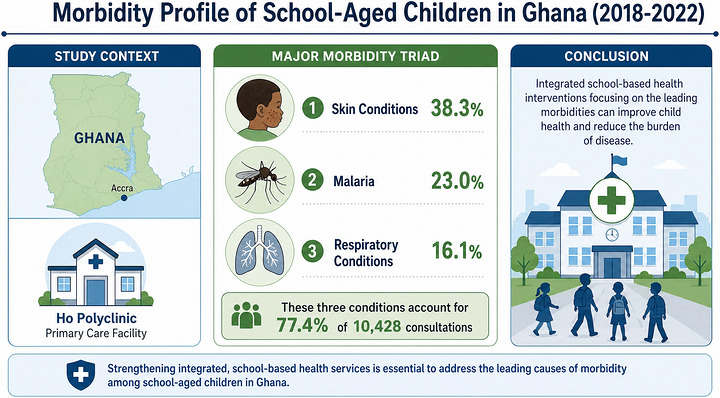

## 1 Introduction

Significant global advancements in child survival over recent decades have been largely driven by targeted interventions for children under 5 years, including expanded immunization programs, insecticide‐treated net distribution for malaria prevention, and nutritional supplementation [1, 2]. This strategic focus has yielded measurable success in reducing under‐five mortality. Consequently, the health needs of school‐aged children (5–17 years) a demographic constituting approximately a quarter of the population in many sub‐Saharan African countries have remained comparatively overlooked in mainstream public health policy and research [3, 4].

This neglect stems from a prevailing, yet flawed, assumption of reduced biological vulnerability in older children. However, a growing body of evidence challenges this notion, demonstrating that morbidity in school‐aged children has direct and consequential impacts on cognitive development, educational attainment, and long‐term socioeconomic potential [5, 6]. School‐aged children face a distinct epidemiological transition, encountering a unique set of health risks that include a high burden of communicable diseases, early manifestations of noncommunicable conditions, and the consequences of environmental and social exposures [7]. In Ghana, children aged 5–17 years represent a substantial population segment of approximately 6.8 million individuals [8].

Although national policy frameworks, such as the Child Health Policy, explicitly target the under‐five demographic, there exists no equivalently structured, nationwide health intervention package for school‐aged children [9].

Ghana's School Health Education Programme (SHEP), established in 1992, mandates health screening, health education, and referral coordination for school‐aged children. However, implementation evaluations reveal persistent gaps: A 2019 assessment in the Volta Region found that only 42% of public schools conducted the recommended annual health screenings, and fewer than 15% had functional linkages with nearby health facilities. In the Ho municipality specifically, SHEP activities are largely donor‐dependent and fragmented, with no systematic capture of morbidity data to guide intervention planning. This aligns with the study conducted by Adomako Gyasi et al., reported that, resource constraints, lack of parental and community participation, and issues with management, leadership, governance, and politics were barriers to implementing school‐based health programs in Ghana [10]. This policy‐implementation gap underscores the need for facility‐level data of the kind our study provides.

Although Ghana operates a SHEP under the Ghana Education Service, its implementation has been inconsistent, focusing predominantly on preventive interventions such as immunization, deworming, and visual screening, with limited integration of curative clinical services for common morbidities such as skin conditions, malaria, or respiratory illnesses. In the Ho municipality, SHEP activities exist but are not systematically evaluated, and no program specifically targets the morbidity triad identified in this study.

This policy gap is exacerbated by a parallel deficit in local epidemiological data; the patterns of disease, their prevalence, and determinants within this age group in Ghana remain inadequately documented and largely inferred from data on younger children or adults.

This study, therefore, sought to address this evidence gap by conducting a comprehensive analysis of morbidity patterns among school‐aged children attending a major primary care facility in southern Ghana. Specifically, the study aimed to (a) describe the spectrum and relative frequency of diseases diagnosed in children aged 5–17 years over a 5‐year period, and (b) investigate potential associations between these leading diagnoses and key demographic variables, namely, age and sex. The findings are intended to generate actionable insights to guide the development of targeted interventions and inform policy discussions aimed at improving health outcomes for this critical but underserved demographic in Ghana and similar contexts.

## 2 Methods

### 2.1 Study Design and Setting

A retrospective cross‐sectional study using routine health records was conducted at the Ho Polyclinic, a primary care facility in the Ho municipality, Volta Region of Ghana (Figure 2 for a map showing clinic location within the region). The clinic, originally established in 1952 as a leprosarium, has evolved into a comprehensive primary care center with a historical reputation for dermatological care. The design is appropriate for describing morbidity patterns and generating hypotheses for future research. The study period (2018–2022) was selected to capture five full years of data, including the pre‐COVID‐19 period (2018–2019), the peak pandemic year (2020–2021), and the endemic phase (2022), allowing for assessment of temporal stability. Ho Polyclinic serves as a primary referral center for 12 community health planning and services (CHPS) zones and four health centers within the Ho Municipal Health Directorate.

The facility records approximately 18,000–22,000 outpatient consultations annually, with an average of 1800–2100 consultations per month. Of these, school‐aged children (5–17 years) constitute approximately 14%–17% of total visits. The outpatient department operates 7 days per week (Monday–Sunday) providing a‐24‐h service. The Ho Polyclinic is staffed by one medical officer, a pediatrician, a dermatologist, five physician assistants, and about 100 nurses. Basic laboratory services (malaria rapid diagnostic testing [RDT], urinalysis, stool microscopy, and hemoglobin measurement) and a pharmacy are available on‐site. The clinic serves an estimated catchment population of 87,500 people, encompassing the Ho municipality and surrounding rural districts (Adaklu, Agortime‐Ziope) [11]. The catchment population is predominantly Ewe‐speaking, with livelihoods based on subsistence farming (40% of households), trading (35%), and public sector employment (25%).

The climate is tropical humid with a bimodal rainfall pattern (major rains: April–July; minor rains: September–November). Average annual temperature ranges from 23°C to 32°C. Housing in rural parts of the catchment is predominantly compound‐style with high occupancy densities (mean 2.8 persons per room in rural areas versus 1.9 in urban Ho township), a factor known to influence transmission of skin conditions such as scabies, impetigo, and yaws [12, 13], and respiratory infections.

The Ho municipality has 127 public basic schools (kindergarten through junior high school) and 43 private basic schools, serving approximately 28,500 school‐aged children. The SHEP operates at the municipal level with two dedicated coordinators. Each school is to receive the recommended two visits in a year. Screening activities focus primarily on deworming (albendazole distribution), visual acuity testing, and basic hygiene education. No systematic screening for skin conditions, malaria (RDT‐based), or respiratory morbidities occurs within schools, creating a reliance on facility‐based care as documented in our study.

### 2.2 Study Population and Data Source

The study population comprised children aged 5–17 years who presented for consultation at the outpatient department between January 1, 2018, and December 31, 2022. All included consultations were for symptomatic illness episodes (i.e., children presenting with one or more symptoms such as fever, rash, cough, difficulty breathing, diarrhea, or other complaints). Routine check‐ups, growth monitoring visits, immunization‐only visits (for catch‐up vaccines in older children), and follow‐up visits for previously diagnosed chronic conditions were excluded to focus on new acute morbidity episodes. Inclusion criteria were: (a) age 5–17 years at time of consultation, (b) documented primary diagnosis recorded by the attending clinician, (c) complete demographic data (age, sex, residence). Exclusion criteria were: (a) incomplete records (missing diagnosis or key demographic variables), (b) duplicate entries for the same consultation episode, (c) visits for medication refills without a new diagnosis, (d) routine well‐child visits without a specific illness complaint. Data were extracted from the paper‐based outpatient morbidity registers.

### 2.3 Variables and Data Collection

A standardized data extraction form was designed using KoboToolbox. Trained research assistants extracted the following variables for each unique visit: date of visit, age, sex, residence (distance band), and primary clinical diagnosis. Diagnoses were recorded after laboratory and radiological investigations and entered by the attending clinician.

### 2.4 Data Analysis

Data were cleaned and analyzed using Stata software version 17.0. Descriptive statistics summarized demographic characteristics and morbidity frequencies. The chi‐square (*χ* ^2^) test was used to assess associations between demographic variables (age group, sex) and the three most frequent diagnostic categories. A *p* value <0.05 was considered statistically significant. Results are presented as frequencies (percentages).

## 3 Results

### 3.1 Demographic Characteristics of Health‐Seeking Children

Over the 5‐year period (2018–2022), 10,428 consultations involving school‐aged children (5–17 years) were recorded at Ho Polyclinic. The mean age was 10.3 years (SD ±4.0). Children aged 5–11 years constituted the majority of visits (61.7%, *n* = 6428). There were 5668 female visits (54.4%) and 4760 male visits (45.6%). The gender and age distribution by year is presented in Table 1.

**TABLE 1:** Age and gender distribution of health facility visits by school‐aged children (5–17 years), Ho Polyclinic, 2018–2022.

Variable	2018 (*n*, %)	2019 (*n*, %)	2020 (*n*, %)	2021 (*n*, %)	2022 (*n*, %)	Total (*n*, %)
Age group (years)						
5–11	1264 (64.2)	833 (59.3)	1177 (60.6)	2136 (62.8)	1018 (59.5)	6428 (61.7)
12–14	324 (16.4)	257 (18.3)	360 (18.6)	534 (15.7)	280 (16.4)	1755 (16.8)
15–17	382 (19.4)	314 (22.4)	404 (20.8)	732 (21.5)	413 (24.1)	2245 (21.5)
Gender						
Female	1156 (58.7)	785 (55.9)	1063 (54.8)	1790 (52.6)	874 (51.1)	5668 (54.4)
Male	814 (41.3)	619 (44.1)	878 (45.2)	1612 (47.4)	837 (48.9)	4760 (45.6)
Total visits	1970	1404	1941	3402	1711	10,428

*Note:* Age groups correspond to Ghana's educational levels: 5–11 years (primary school), 12–14 years (junior high school), and 15–17 years (senior high school). This stratification facilitates translation of findings into school‐based intervention planning.

### 3.2 Overall Morbidity Profile

Skin conditions, malaria, and respiratory conditions together accounted for 77.4% (*n* = 8074/10,428) of all diagnoses. The annual distribution of the top 10 diagnoses is shown in Table 2. Skin conditions were the leading diagnosis each year (overall 38.3%), followed by malaria (23.0%) and respiratory conditions (16.1%). Surgical conditions, including trauma, lacerations, fractures, sprains, and sports‐related injuries, accounted for 2.2% (*n* = 233) of consultations—lower than expected for this age group, suggesting potential under‐utilization of facility care for minor injuries.

**TABLE 2:** Annual distribution of the top 10 diagnoses among school‐aged children, Ho Polyclinic, 2018–2022.

Diagnosis	2018 (*n*, %)	2019 (*n*, %)	2020 (*n*, %)	2021 (*n*, %)	2022 (*n*, %)	Total (*n*, %)
Skin conditions	863 (43.8)	592 (42.2)	677 (34.9)	1189 (35.0)	674 (39.4)	3995 (38.3)
Malaria	297 (15.1)	242 (17.2)	606 (31.2)	859 (25.3)	399 (23.3)	2403 (23.0)
Respiratory conditions	323 (16.4)	277 (19.7)	262 (13.5)	600 (17.6)	214 (12.5)	1676 (16.1)
Diarrhoeal diseases	41 (2.1)	56 (4.0)	111 (5.7)	88 (2.6)	119 (7.0)	415 (4.0)
Urinary tract infection	137 (7.0)	51 (3.6)	18 (0.9)	88 (2.6)	22 (1.3)	316 (3.0)
Intestinal worms	12 (0.6)	30 (2.1)	58 (3.0)	109 (3.2)	71 (4.2)	280 (2.7)
ENT conditions	111 (5.6)	35 (2.5)	26 (1.3)	60 (1.8)	36 (2.1)	268 (2.6)
Surgical conditions^a^	94 (4.8)	26 (1.9)	26 (1.3)	61 (1.8)	26 (1.5)	233 (2.2)
Anemia	12 (0.6)	12 (0.9)	37 (1.9)	94 (2.8)	14 (0.8)	169 (1.6)
Peptic ulcer disease	15 (0.8)	31 (2.2)	18 (0.9)	71 (2.1)	27 (1.6)	162 (1.6)
Other conditions^b^	65 (3.3)	50 (3.6)	102 (5.3)	183 (5.4)	111 (6.5)	1511 (14.5)
Total	1970	1404	1941	3402	1711	10,428

^a^ *Surgical conditions* include trauma, lacerations, fractures, sprains, and sports‐related injuries.

^b^ *Other conditions* comprise diagnoses not listed in the top 10. The three most frequent among these were musculoskeletal pain (*n* = 89), eye infections/conjunctivitis (*n* = 76), and dental caries (*n* = 54).

### 3.3 Associations Between Demographic Factors and Top Three Diagnoses

Among school‐aged children attending Ho Polyclinic, skin conditions (38.0%–39.2%), malaria (21.8%–23.6%), and respiratory conditions (15.6%–16.3%) are the three most common diagnoses across all age subgroups (5–11, 12–14, and 15–17 years) and both sexes (female and male). Chi‐square tests reveal no statistically significant associations between age group and any of the three diagnoses (*p* = 0.598, 0.176, and 0.839, respectively). Similarly, no significant sex‐based differences are observed (*p* = 0.611, 0.452, and 0.555, respectively). These null findings indicate that the burden of these leading morbidities is distributed uniformly across the entire school‐aged population, without disproportionate concentration in any specific age or gender subgroup (Table 3).

**TABLE 3:** Association of age group and gender with the three leading diagnoses.

Diagnosis	Age 5–11 years (*n* = 6428)	Age 12–14 years (*n* = 1755)	Age 15–17 years (*n* = 2245)	*p* value (age)	Female (*n* = 5668)	Male (*n* = 4760)	*p* value (sex)
Skin conditions	2442 (38.0%)	673 (38.3%)	880 (39.2%)	0.598	2184 (38.5%)	1811 (38.0%)	0.611
Malaria	1519 (23.6%)	394 (22.4%)	490 (21.8%)	0.176	1290 (22.8%)	1113 (23.4%)	0.452
Respiratory conditions	1037 (16.1%)	274 (15.6%)	365 (16.3%)	0.839	922 (16.3%)	754 (15.8%)	0.555

*Note:* Column percentages use denominators from Table 1 (total consultations per subgroup). *p* values are from chi‐square tests that included all diagnostic categories (not only the three shown).

### 3.4 Monthly and Spatial Patterns

**FIGURE 1:**
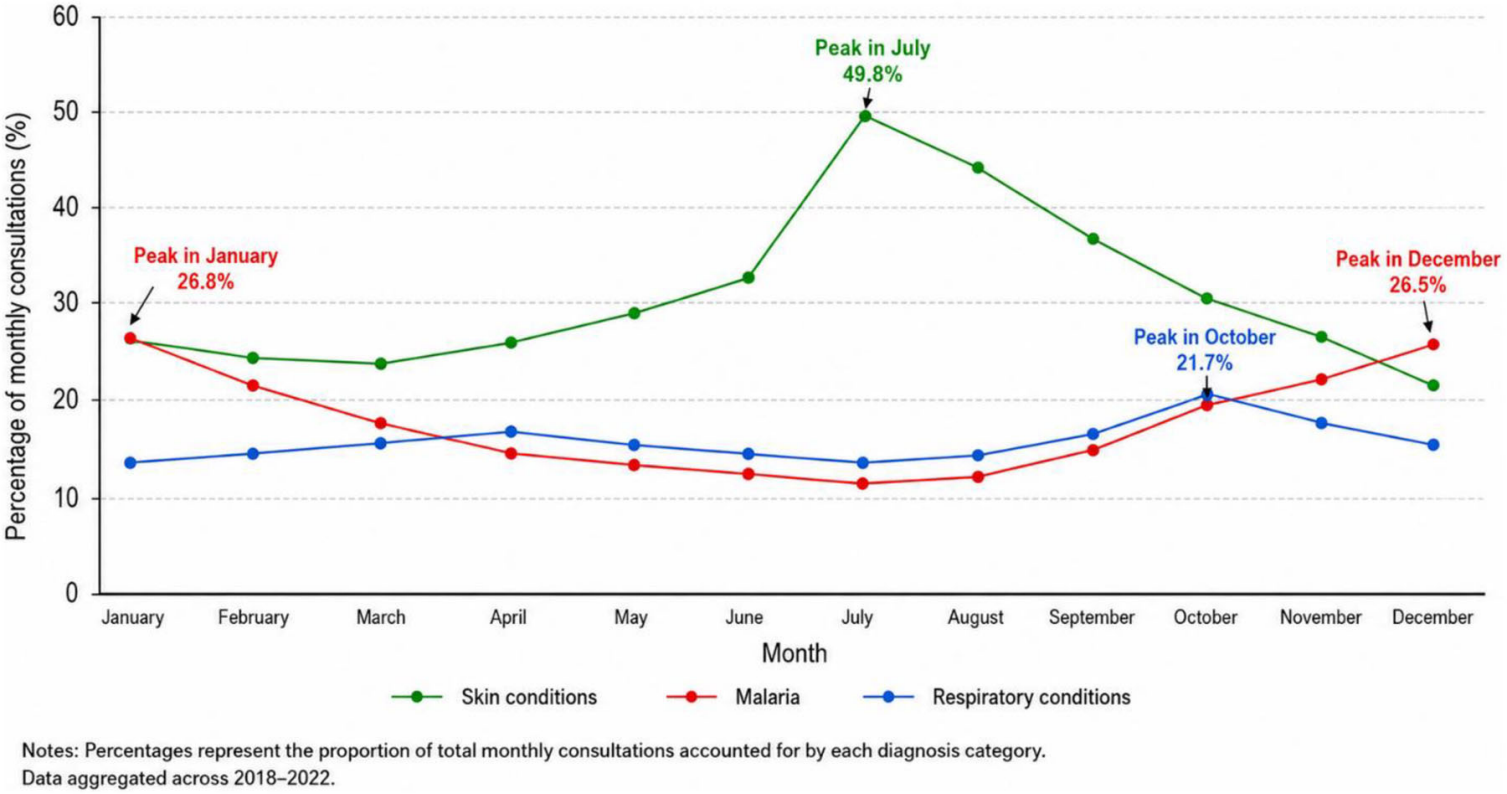
Monthly distribution of the three diagnoses (January–December, aggregated across 2018–2022).

The monthly distribution of the three leading diagnoses is illustrated in Figure 1. Skin conditions peaked in July, accounting for 49.8% of diagnoses recorded during that month. Malaria diagnoses were highest in January (26.8%) and December (26.5%), whereas respiratory conditions increased during October (21.7%).

**FIGURE 2:**
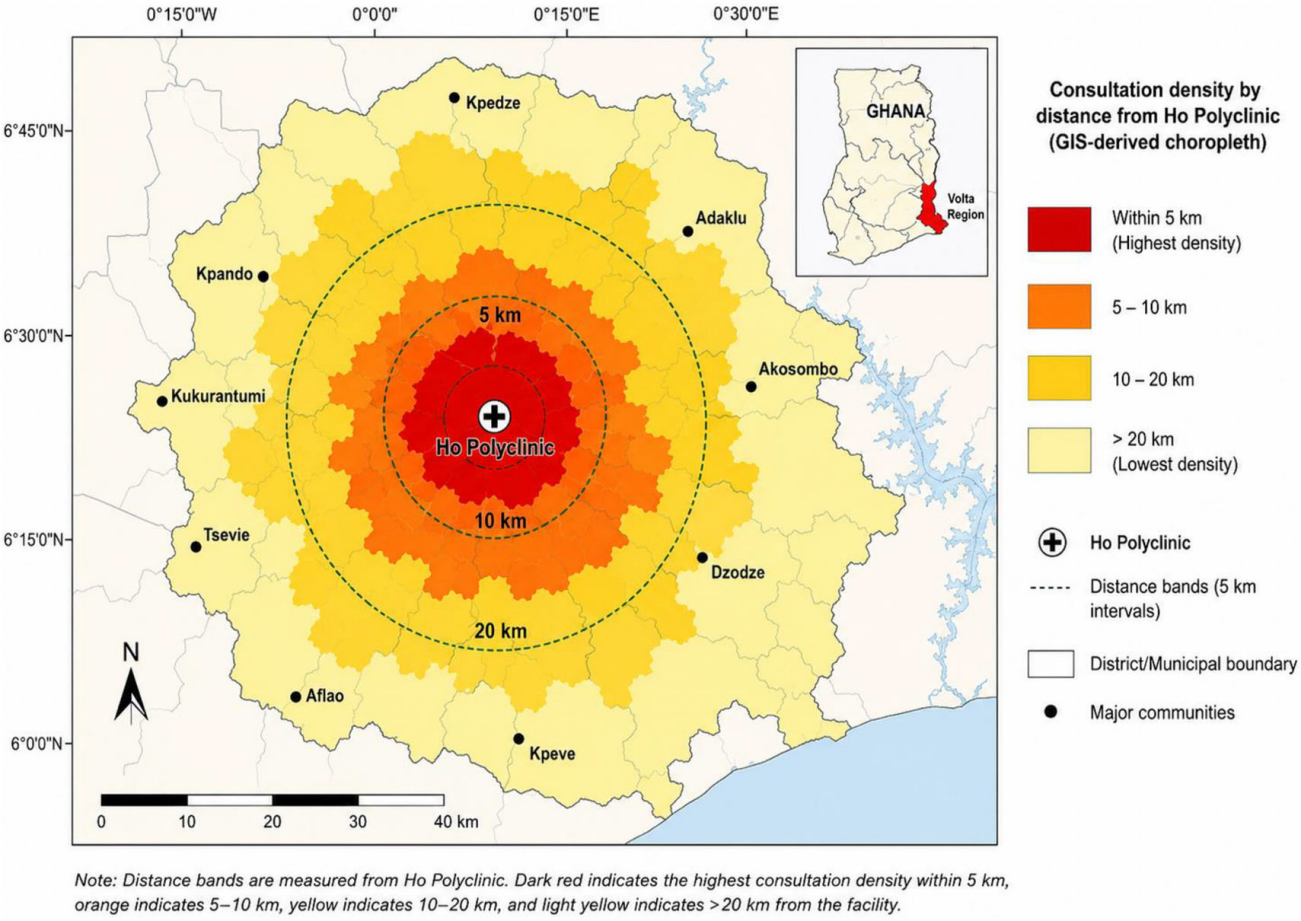
Geospatial distribution of consultations among school‐aged children attending Ho Polyclinic, 2018–2022. GIS, geographic information systems.

Geospatial analysis demonstrated that most consultations originated from communities located near Ho Polyclinic. Overall, 22.3% of children resided within 5 km of the facility, whereas 36.0% lived between 5 and 10 km away. Consultation density progressively declined with increasing distance from the facility. The geographic information systems (GIS)‐derived choropleth map in Figure 2 illustrates the spatial concentration of consultations by distance band, with the highest density observed within the 5 km radius.

## 4 Discussion

This retrospective study provides a detailed epidemiological profile of the leading morbidities among school‐aged children (5–17 years) attending a primary care facility in southern Ghana. The analysis of over 10,000 consultations across 5 years reveals a clear and consistent pattern: a triad of skin conditions (38.3%), malaria (23.0%), and respiratory conditions (16.1%) collectively accounts for over three‐quarters (77.4%) of the disease burden in this population. Critically, the prevalence of these conditions showed no statistically significant variation by age group or gender, suggesting they represent universal health risks for all school‐aged children in this setting. These findings underscore a significant gap in targeted health interventions, which traditionally focus on children under five and pregnant women, and provide a strong evidence base for reorienting public health strategies in the Ho municipality and similar contexts.

The finding that skin conditions constitute the leading cause of presentation, representing nearly two‐fifths of all diagnoses, is the most striking result of this study. This must be interpreted within the specific context of the Ho Polyclinic, which was historically established as a leprosarium and maintains a strong reputation for dermatological care [14]. The sheer volume of cases (*n *= 3995) indicates a substantial, real burden of dermatological disease requiring clinical attention.

In our study, the most frequently diagnosed skin conditions were tinea (37.0% of all skin diagnoses, accounting for approximately 14.2% of total consultations), allergic dermatitis (20.6%), and impetigo (15.3%). This profile of dermatological conditions differs notably from findings reported in other resource‐limited settings across sub‐Saharan Africa.

For instance, in rural Guinea, infectious dermatoses—particularly scabies and ringworm—predominate, accounting for 94.6% of skin pathologies among 615 patients [15]. Although fungal infections, such as tinea, are common in that context, the high burden of scabies and the relative prominence of allergic conditions differ from the profile observed in our cohort. Similarly, a study involving 603 patients in Senegal reported a high prevalence of bacterial infections (impetigo accounting for 57% of bacterial dermatoses) and fungal infections (29.41%), alongside a substantial burden of allergic dermatoses, including atopic dermatitis (21.72%) and contact eczema (24.7%) [16]. In that setting, impetigo and allergic conditions represented a larger share of diagnoses compared to our findings, whereas tinea appeared less dominant.

On a broader scale, a retrospective study of 4188 patients at AIIMS in Raebareli, Uttar Pradesh, India, found that infections—particularly fungal infections, which constituted 78% of all infectious cases—represent the most prevalent dermatological conditions across all age groups, with dermatitis being a major concern among adults [17]. This aligns with our observation that infections, especially tinea, are a leading cause of dermatological morbidity. However, the relative contributions of bacterial versus fungal infections, as well as the prominence of allergic dermatitis, appear to vary significantly across populations, likely reflecting differences in climate, hygiene practices, socioeconomic conditions, and access to healthcare.

Taken together, these comparisons underscore the importance of context‐specific epidemiological data to guide diagnostic and therapeutic priorities. Our findings contribute to this body of evidence by highlighting tinea as the predominant condition in our setting, with allergic dermatitis and impetigo representing secondary but still significant burdens.

Similar study conducted in Korle‐Bu Teaching hospital, Accra revealed, specific dermatological conditions, including atopic dermatitis (8.4%), acne vulgaris (5.3%), and scabies (5.1%), were frequently observed, exhibiting significant age‐specific prevalence. Atopic dermatitis predominated in pediatric cohorts (infants: 31.7%, children: 30.0%, and adolescents: 14.9%), whereas acne vulgaris was most common in young adults (12.0%), irritant contact dermatitis in adults (6.9%), and lichen planus in the senior population (9.9%). These findings highlight the diverse dermatological burden across age groups and underscore the imperative for enhanced dermatology education and training at the primary care level in Ghana to improve diagnostic precision and optimize patient management strategies [18].

In Ghana and similar low‐resource settings, such preventable and treatable skin infections are major contributors to school absenteeism, stigma, and reduced quality of life among children [19]. Therefore, although our prevalence estimate may be inflated, it successfully flags a neglected area of school health that demands intervention. Integrated school health programs should emphasize personal hygiene, provision of basic treatment (e.g., antifungal and antibacterial creams), and community awareness to reduce the burden of skin conditions. The inherent *referral bias* in this study could elevate the proportional morbidity seen in our facility‐based data compared to the true community prevalence. However, dismissing the finding based solely on this bias would be a missed opportunity for public health action [20, 21].

Despite global and national progress in malaria control, our data confirm that malaria remains a leading cause of morbidity among school‐aged children in Ho, responsible for nearly a quarter of all visits. This is buttressed by Isezuo et al. that malaria accounts for the top morbidity among children aged above 5 years [22]. Our finding is particularly significant as it highlights a potential gap in the malaria control continuum. Ghana's national strategy, aligned with WHO recommendations, heavily emphasizes protection for children under five and pregnant women through mechanisms like seasonal malaria chemoprevention (SMC) and insecticide‐treated bed net (ITN) distribution campaigns [23]. Our results suggest that children aged 5–17 may represent a residual reservoir of infection, possibly due to waning immunity and increased environmental exposure without the benefit of targeted chemoprevention [24]. The absence of a significant age‐based association within the 5–17 year cohort further indicates that this risk is sustained throughout the school‐age years. This calls for a revision of malaria intervention strategies to be more inclusive of school‐age children.

Our finding that malaria accounts for 23.0% of consultations among school‐aged children is consistent with a growing body of literature documenting persistent malaria risk in this demographic. A study conducted by Pinchoff et al. [25] reported an 8.83 times higher odds of malaria positivity in school‐aged children compared to adults in a high‐transmission region of Zambia. Similarly, Walldorf et al. [26] found that children aged 6–15 years in Malawi had higher odds of both asymptomatic infection (OR = 4.2) and clinical malaria (OR = 4.8) compared to younger children. A recent study by Nundu et al. in the Democratic Republic of Congo explicitly called for integrating school‐aged children into national malaria control programs after documenting that this group bears a disproportionate share of the residual transmission burden [27]. Our data from Ghana add to this evidence base, confirming that the pattern holds across diverse sub‐Saharan African settings.

The observed peak in malaria diagnoses during December (26.5%) and January (26.8%) warrants discussion of local transmission dynamics. The Volta Region experiences a bimodal rainfall pattern, with major rains from April to July and minor rains from September to November. Malaria transmission typically peaks approximately 4–6 weeks after the major rains (August–September) and again after the minor rains (December–January). The December–January peak observed in our study aligns with the post‐minor‐rain transmission surge but may also reflect increased healthcare‐seeking behavior during the school vacation period (mid‐December through early January), when children are more likely to be taken to health facilities for febrile illnesses that might otherwise be managed at school or home. This interaction between biological transmission dynamics and health‐seeking behavior should be considered in interpreting seasonal patterns from facility data.

A more inclusive approach is essential to comprehensively address the persistent vulnerability of this significant population segment, thereby enhancing overall public health outcomes. Evidence supports the efficacy and feasibility of school‐based interventions, such as intermittent preventive treatment in schools (IPTsc) and targeted distribution of ITNs through educational institutions, which could significantly reduce the burden in this demographic and contribute to broader community‐wide transmission reduction [28, 29].

Respiratory conditions, the third leading cause of morbidity, reflect a consistent challenge in pediatric populations in low‐ and middle‐income countries (LMICs). The types of conditions captured (e.g., upper respiratory tract infections, bronchitis, and pneumonia) are influenced by multiple factors prevalent in the Ho municipality, including household air pollution from solid fuel use, seasonal variations, and urban air quality [30, 31]. The introduction of the 13‐valent pneumococcal conjugate vaccine (PCV13) in Ghana has undoubtedly reduced the incidence of severe pneumococcal disease in children under five [32, 33]. However, our data suggest that school‐aged children remain vulnerable to a high burden of non‐severe respiratory infections. Furthermore, studies indicate high rates of pneumococcal carriage with non‐vaccine serotypes in older children in Ghana, perpetuating transmission and disease [34]. This underscores the need for continued efforts beyond vaccination, including public health campaigns on respiratory hygiene, improved ventilation in homes and schools, and policies to address ambient air pollution.

Our study found that respiratory conditions accounted for 16.1% of all outpatient consultations in Ghana, a proportion that mirrors estimates from other primary care settings in sub‐Saharan Africa. Respiratory diseases represent a major yet under‐recognized burden in the region. Asthma prevalence ranges from 5.7% to 20.3%, with higher rates observed in urbanized areas [35]. In South Africa, childhood asthma prevalence reaches 13%, associated with substantial morbidity [36]. Chronic obstructive pulmonary disease (COPD) is increasingly common, driven by tobacco smoking and exposure to biomass fuel, which affects approximately 90% of rural households [35]. Concurrently, respiratory infections remain leading causes of morbidity and mortality across sub‐Saharan Africa [37].

Despite this burden, critical evidence gaps persist. A qualitative study across five countries (Kenya, Malawi, Sudan, Tanzania, and Uganda) involving 109 stakeholders found that chronic respiratory diseases are consistently “underdiagnosed, under‐reported and underfunded,” largely due to weak diagnostic capacity and inadequate health information systems [38]. Researchers have explicitly called for high‐quality, context‐specific epidemiological data to inform health system planning [39]. The observed increase in cases during October and November may align with seasonal changes in humidity and allergen levels, warranting further investigation for targeted preventive messaging [40].

A key insight from this study is the observed lack of a statistically significant association between the three most prevalent conditions (skin infections, malaria, and respiratory illnesses) and specific age subgroups (5–11, 12–14, and 15–17 years) or gender within the school‐aged cohort. This null finding is particularly informative for public health planning, as it indicates that the burden of these conditions is not disproportionately concentrated within a particular demographic segment of school‐aged children. Consequently, interventions designed to mitigate these prevalent health issues should be universally accessible to all school‐aged children, thereby promoting equity in service delivery. This evidence strongly supports the implementation of integrated, school‐based health programs capable of delivering comprehensive preventive education, screening, and basic treatment to all pupils without discrimination, ensuring maximal coverage and public health impact [41].

### 4.1 Limitations

The interpretations of this study must be considered in light of several limitations. First, as a retrospective review of records from a single facility with a dermatological specialization, the findings are subject to significant referral bias and may not be generalizable to the community prevalence or to other health facilities in Ghana. Second, the cross‐sectional nature of the visit‐based data precludes conclusions about disease incidence or causal relationships. Third, the lack of data on socioeconomic status, schooling, and detailed environmental exposures limits our ability to explore deeper determinants of the observed morbidity patterns.

Fourth, the single‐facility design without a comparator nonspecialized clinic limits our ability to separate true community morbidity from facility‐specific referral patterns. Future multisite studies should include facilities with varied reputational profiles to establish community‐representative estimates. Fifth, the low proportion of trauma/sports injuries (2.2%) likely reflects under‐ascertainment due to home management or school first‐aid rather than true low incidence. Finally, the absence of laboratory confirmation for many respiratory and skin diagnoses (which were based on clinical examination alone) may introduce misclassification bias, though this reflects real‐world diagnostic practices in primary care settings.

Finally, the use of linear distance bands is a crude proxy for healthcare access; more nuanced GIS analyses could yield deeper insights.

## 5 Conclusion and Recommendation

This study provides facility‐based evidence among school‐aged children in the Ho municipality who consistently presented preventable and treatable conditions such as skin infections, malaria, and respiratory illnesses. Ghana can address this critical gap in its disease control efforts, improve educational outcomes, and make significant strides towards achieving universal health coverage for all age groups by 2030.

The uniformity of this burden across age and gender subgroups within 5–17 years argues for broad, inclusive intervention strategies. To translate these findings into action, we recommend that the Ho Municipal Health Directorate, in collaboration with the Ghana Education Service, prioritizes the following:

- Implement regular school health screenings with components for skin health checks, malaria RDT during high‐transmission seasons, and respiratory health education.
- Intensify and evaluate the feasibility and impact of interventions such as IPTsc or targeted insecticide‐treated bed net (ITN) distribution through schools.
- Scale up hygiene promotion (handwashing, personal care) in schools and communities, and investigate interventions to reduce respiratory diseases.

## Author Contributions

**Anthoinette Adesope Ekuban** and **Frank Baiden** participated in the conception, design of the study, data management, analysis and drafting of the manuscript. All the authors participated in reading the final version of the manuscript.

## Funding

The authors have nothing to report.

## Conflicts of Interest

The authors declare no conflicts of interest.

## Ethics Statement

Ethical approval was granted by the Ghana Health Service Ethics Review Committee (GHS‐ERC: 005/03/24). Administrative approval of this study was also granted by the management of Ho Polyclinic. The study used anonymized secondary data so written informed consent was not applicable.

## Transparency Statement

The lead author affirms that this manuscript is an honest, accurate and transparent account of the study being reported; that no important aspects of the study have been omitted; and that any discrepancies from the study as planned (and, if relevant, registered) have been explained.

## Data Availability

All relevant data are presented within the manuscript. The dataset used for the analysis can, however, be obtained from the corresponding author upon reasonable request. The corresponding author has full access to all the data in this study and takes complete responsibility for the integrity of the data and the accuracy of the data analysis.
